# Bibliometric Analysis of The Global Research Trends on The Application of Tamoxifen in The Treatment of Breast Cancer Over The Past 50 Years

**DOI:** 10.21315/mjms-06-2024-452

**Published:** 2025-02-28

**Authors:** Ninie Nadia Zulkipli, Rahimah Zakaria, Wan Rohani Wan Taib

**Affiliations:** 1 School of Biomedicine, Faculty of Health Sciences, Universiti Sultan Zainal Abidin, Terengganu, Malaysia; 2 School of Medical Sciences, Universiti Sains Malaysia, Health Campus, Kelantan, Malaysia

**Keywords:** bibliometric analysis, breast cancer, Harzing’s Publish or Perish, Scopus, tamoxifen, VOSviewer

## Abstract

GLOBOCAN 2022 reported that breast cancer is a primary contributor to the incidence and mortality rates of cancer in women. The very high and low Human Development Index (HDI) tiers are the primary contributors to the highest incidence and mortality rates, respectively. This study aimed to provide a comprehensive landscape overview of trends, dynamics, and research hot spots on the application of tamoxifen in treating breast cancer over the past 50 years. We retrieved data from the Scopus database, spanning from 1973 to 2022. We utilised Microsoft Excel, Harzing’s Publish or Perish, and VOSviewer to perform exhaustive analyses, including the publication trend, co-authorship, co-citation, co-occurrence of authors, organisations, countries, and keywords. A total of 3,721 publications fit the inclusion and exclusion criteria. Jordan VC was the most prominent author, making substantial contributions to the research topic. Erasmus MC was the most prolific organisation, while the Swiss Group for Clinical Cancer Research exhibited the most robust international collaboration. The United States and the United Kingdom consistently have the highest publication, TLS, and *h*- and *g*-indices. Using keyword co-occurrence analysis, we identify adjuvant endocrine therapy, postmenopausal, EGFR, HER2, and autophagy as research hot spots. For the past five decades, the research output of the application of tamoxifen in breast cancer treatment has exhibited an upward trend. This endeavour provides a crucial reference for researchers to direct greater attention toward research hot spots in the hope that it will improve breast cancer patients’ treatments and, consequently, increase their survival rate.

## Introduction

The Global Cancer Observatory (GLOBOCAN) 2022 estimated approximately 20 million new cancer cases globally, with 9.7 million cancer-related fatalities. Female breast cancer is the second most commonly diagnosed cancer after lung cancer, with 2.3 million new cases, representing 11.5% of all cancer cases (1). This marks a 0.2% reduction compared to GLOBOCAN 2020. Furthermore, with 665,683 deaths, female breast cancer ranks as the fourth leading cause of cancer-related death. The Human Development Index (HDI), a composite measure that includes health, education, and income indicators, was first introduced in 1990. Research has found that the HDI correlates with the incidence and mortality rate of breast cancer (2, 3).

Breast cancer is categorised into four molecular subtypes based on immunohistochemistry (IHC): i) luminal A (ER+, PR+, HER2-, Ki67 < 20%); ii) luminal B (ER+, PR+ and HER2+ and Ki67≥ 20%); iii) HER2-enriched (ER−, PR−, HER2+); and iv) basal-like (triple-negative: ER−, PR−, HER2) (4). Additionally, there is a fifth subtype, normal-like, which shares similarities with other IHC subtypes but has distinct gene expression patterns and a poor prognosis (5). Understanding these intrinsic subtypes is crucial, as they reflect the heterogeneity in biological activity and gene expression, serving as prognostic indicators and aiding in identifying optimal therapeutic approaches (6). The ER/PR positive subtypes (luminal A and luminal B) dominate globally, comprising 70% of all reported cases. Luminal A and B account for approximately 40%–60% and 20%–30% of cases, respectively (7, 8). Hormone therapy is typically recommended for patients with luminal breast cancer, though adjuvant chemotherapy is more effective in a minority of these cases (9).

Current endocrine therapies include selective oestrogen receptor modulators (SERMs), aromatase inhibitors (AIs), and selective oestrogen receptor downregulators (SERDs) (10). Tamoxifen [trans-1-(4-b-dimethylaminoethoxyphenyl)-1,2-diphenylbut-1-ene], a non-steroidal SERM, has revolutionised breast cancer treatment and prevention since its US Food and Drug Administration (FDA) approval in 1977 (11). Despite a decrease in its use for advanced breast cancer treatment, tamoxifen remains widely used for prevention in both pre-and postmenopausal women (11, 12). Given the extensive body of literature on tamoxifen’s application in breast cancer treatment, this study aims to analyse and review the bibliographic data and present global research trends over the past 50 years. To our knowledge, this is the first bibliometric analysis of tamoxifen application in breast cancer treatment, focusing on trends in publication outputs, citations, analysis of authors, collaboration between organisations and countries, and keyword co-occurrence.

## Methods

### Data Acquisition and Search Strategy

**Figure 1:**
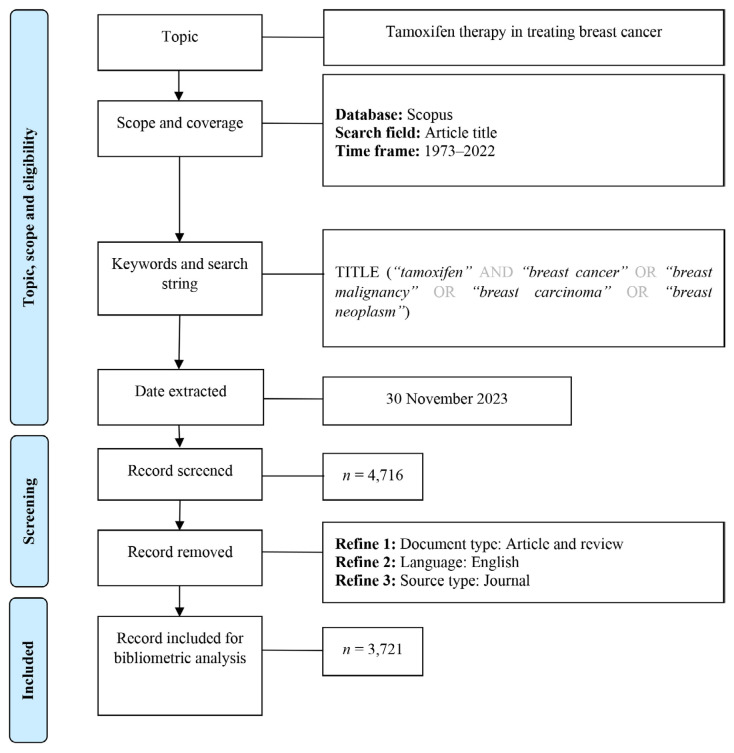
Flow diagram of search strategy (13)

Data was retrieved from the Scopus database and imported in.xls,.ris, and.csv files for analysis using Microsoft Excel 2019, Publish or Perish 8, and VOSviewer, respectively. Our search string, “tamoxifen” AND (“breast cancer” OR “breast malignancy” OR “breast carcinoma” OR “breast neoplasm”), covers publications from 1973 to 2022. We included original research and reviews written in English and published in scholarly journals. A total of 3,721 documents were identified and downloaded for further analysis. Figure 1 illustrates the process of data retrieval, collection, and the filters implemented in this study.

### Data Cleaning

Data cleaning is a crucial step in ensuring the accuracy and reliability of analytical outputs. This study cleaned data before conducting several analyses, including: i) author and cocitation author analysis; ii) co-authorship analysis of organisations; and iii) co-occurrence analysis of author keywords. The primary goal of data cleaning in this study was to standardise authors’ names, organisational affiliations, and author keywords to ensure consistency and precision in the analyses.

The data cleaning process involved scrutinising the Scopus author identifier obtained from the scopus.csv file to standardise author names. This was done to align each author’s name in the analysis with the name used in the author profiles on Scopus, particularly in instances where an author’s name appeared in multiple different written forms. Moreover, the procedure for data cleaning concerning organisational affiliations was carried out using the Google search engine. The formal and recognised names of organisational affiliations were consistently applied throughout the analysis. Additionally, data cleaning prior to conducting co-occurrence analysis of author keywords entailed the standardisation of synonymous keywords, such as converting “breast carcinoma” and “breast neoplasm” into the unified term “breast cancer”. A specific thesaurus.csv file was prepared for different analyses. This file, which contained the finalised version of author names, organisational affiliations, or author keywords, was utilised during the analysis step in VOSviewer.

### Data Analysis

The frequencies and percentages of the published items were calculated using Microsoft Excel 2019 in this investigation. The software tool Harzing’s Publish or Perish was used to calculate citation metrics such as total citations, citations per year, citations per cited paper, and *h*- and *g*-indices. VOSviewer, a software application developed by Van Eck and Waltman in 2010, facilitates creating, visualising, and exploring maps derived from diverse data sources (network, bibliographic, and text). VOSviewer is an intuitive software application with enhanced graphic quality, flexibility, and simplicity (14). This software application incorporates data from reputable and established bibliometric databases, including Scopus (15). VOSviewer can conduct various analyses, such as bibliographic coupling, citation, co-occurrence, co-authorship, and co-occurrence. These analyses are intricately connected to three distinct categories of maps: network-, overlay-, and density visualisations (14).

The node indicates a distinct parameter, such as authors, organisations, countries, keywords, documents, sources (journals), and references, which are determined through analytical selection. Links, documents, total link strength (TLS), citations, and normalised citations are some of the properties weighted to reflect the sizes of the nodes. The hue of the node is determined by the average publication year, average citations, and average normalised citations. Several analyses were performed in this study, including: i) citation metrics analysis; ii) co-citation of authors; iii) co-authorship of organisations and countries; and iv) co-occurrences of author keywords.

## Results

### Citation Metrics Analysis

The citation metrics analysis spanning from 1973 to 2022 underscores the profound impact and enduring research interest in tamoxifen’s application for treating breast cancer. Researchers have published 3,721 papers on the subject, accumulating a remarkable 220,507 citations, averaging 4,410.14 per year, thus emphasising its ongoing relevance in clinical and academic settings. Collaboration was evident, with an average of 5.99 authors per paper. The *h*-index of 197 and *g*-index of 362 further highlight tamoxifen’s substantial impact on breast cancer therapy research, measuring both productivity and citation impact.

### Global Trends in Publication Outputs and Citations

**Figure 2:**
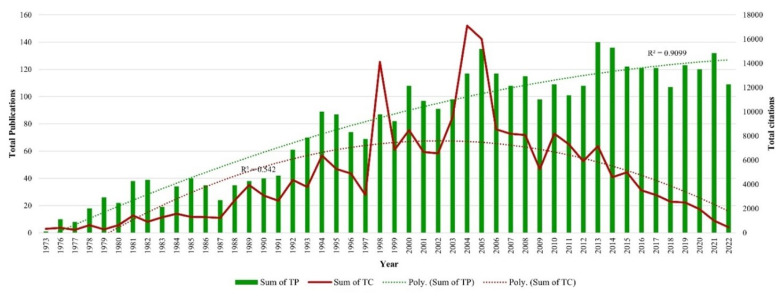
Publications and citations trends in publications related to the research on tamoxifen application in treating breast cancer from 1973 to 2022

Research on the application of tamoxifen for treating breast cancer began in 1973 and continues to be active through 2022 (Figure 2). The first publication titled “Anti-oestrogen therapy for breast cancer: a trial of tamoxifen at two dose levels” was a significant milestone in this field (16). Over the years, the number of publications on tamoxifen has steadily increased, following a polynomial pattern with an R^2^ value of 0.9099. The publication trends by decade are as follows:1973–1982: TP = 162 or 4.35%; 1983–1992: TP = 368 or 9.89%; 1993–2002: TP = 854 or 22.95%; 2003–2012: TP = 1,106 or 29.72%; 2013–2022: TP = 1,231 or 33.08%. Most publications were in 2013 (140), while the lowest was in 1973 (1). Total citations also follow a polynomial pattern (R^2^ = 0.542), although there has been a consistent decline in recent years. The highest number of citations was recorded in 2004 (17,109), and the lowest in 1977 (252).

### Author and Co-citation Author Analysis

**Figure 3:**
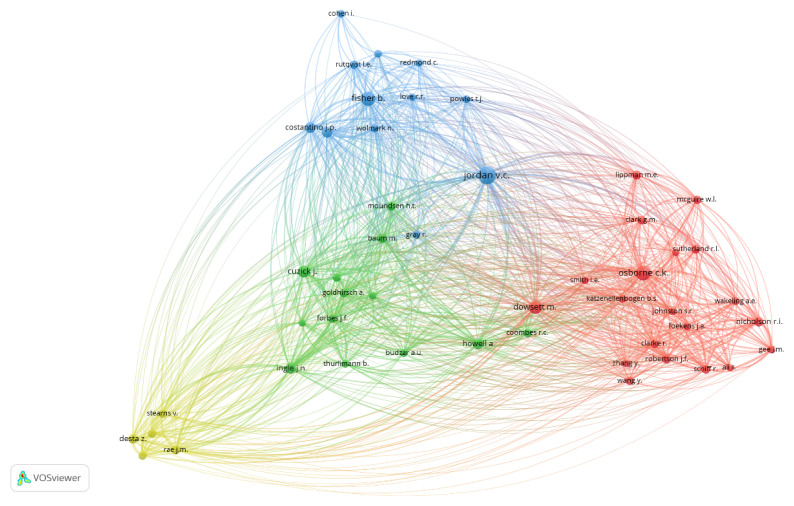
The network visualisation of the top 50 authors with the greatest TLS (*T* ≥ 382). The size of each node represents the weight of TLS, the line between nodes indicates the collaboration among authors, and the specific colour represents each cluster. The red, green, blue, and yellow colours represent Cluster 1, Cluster 2, Cluster 3, and Cluster 4, respectively. *T* is the minimum number of citations by an author

One hundred twenty-three thousand one hundred sixty-four authors contributed to publications on tamoxifen application in breast cancer treatment. Jordan VC led with 62 publications, followed by Dowsett M (50) and Stål O (43) (Table 1). Figure 3 shows the identification and visualisation of 50 authors with at least 382 citations, each using VOSviewer for co-citation analysis. We found four distinct groups, with Jordan VC having the highest TLS (38,818), followed by Osborne CK (28,474), and Dowsett M (25,684). The authors with the lowest TLS include Wang Y (5,377), Zhang Y (4,445), and Cohen I (2,946) (Table 2). Each author in a distinct cluster is connected to the others; no author is isolated.

**Figure 4:**
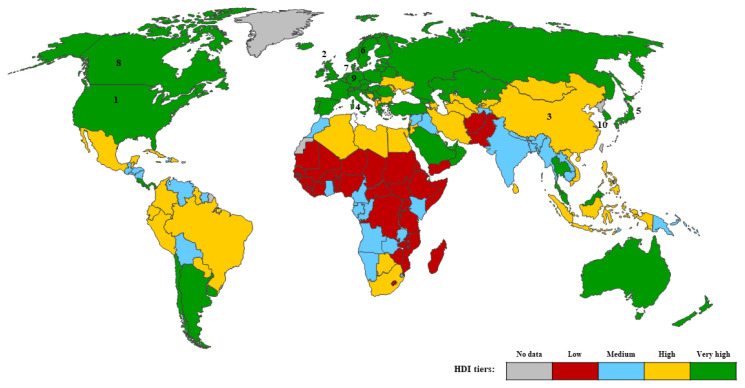
Geographical distribution map of the top 10 countries based on ranking by the TP metric

Jordan VC, Dowsett M, Cuzick J, and Howell A are not only in the top 10 most productive authors but also among the top 10 authors with the highest TLS value. Tables 1 and 2 show that every author in these two categories is from a country that falls within the very high HDI tier. In addition, except Switzerland, all of these countries are ranked among the top 10 productive countries actively researching tamoxifen’s application for treating breast cancer (Figure 4).

### Organisations and International Collaboration Analysis

A total of 160 organisations investigates the therapeutic application of tamoxifen for breast cancer. The top ten prestigious organisations are affiliated with countries as follows: Sweden (4), the United States (3), the United Kingdom (2), and the Netherlands (1), as detailed in Table 3. Erasmus MC, The University of Texas MD Anderson Cancer Centre, and Karolinska Universitetssjukhuset have the highest publication output. The National Cancer Institute has made the most significant contribution, with 349.35 citations per paper, followed by Dana-Farber Cancer Institute (279.90 citations) and Cancer Research UK (263.08 citations) (Table 3).

Next, these 160 organisations were further analysed for the collaboration between the other organisations. Based on this analysis, Switzerland is a significant contributor to the research on the application of tamoxifen in treating breast cancer, followed by the United States, Italy, and Taiwan. The Swiss Group for Clinical Cancer Research (SAKK), Dana-Farber Cancer Institute, and the Frontier Science and Technology Research Foundation have the highest number of collaborations, represented by TLS value. Furthermore, the National Cancer Institute, British Columbia Cancer Agency, and Memorial Sloan-Kettering Cancer Center were the top three organisations with a substantial average number of citations (Table 4). The average citation statistic serves as an indicator of evaluating the impact, quality, and reputation of an organisation. Therefore, it can be inferred that these three organisations possess superior quality research and provide substantial contributions to tamoxifen’s administration in treating breast cancer patients.

### The Contributions of Countries to Global Publications

The retrieved documents were authored by researchers from 88 different countries. Figure 4 illustrates the top ten countries with their HDI that made the most substantial contributions to publications. Notably, all the countries have a very high HDI, except for China, which has a high HDI (17). This demonstrates the strong correlation between high HDI and research productivity. Table 5 shows the TP data for the top 10 most productive countries.

**Figure 5:**
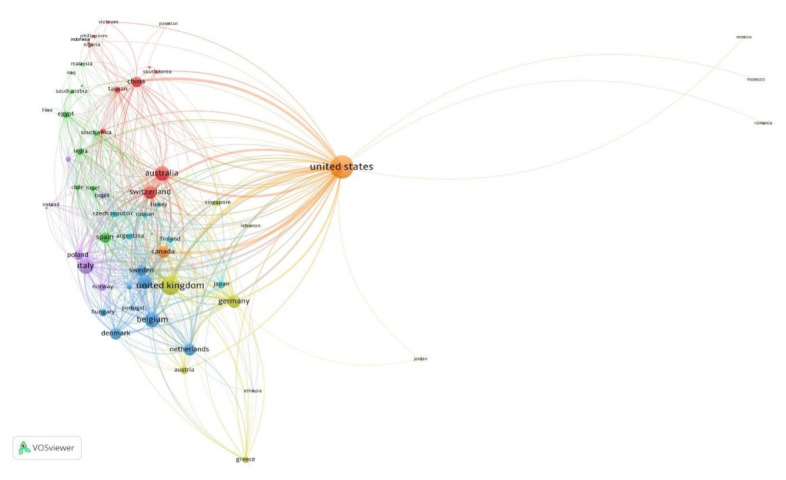
Network visualisation map of international collaboration among countries with a minimum productivity of two (2) documents. The node size and thickness of the connecting line represent the TLS and the strength of collaboration between the two countries

Figure 5 presents a VOSviewer-generated collaboration network map of countries with a minimum productivity of two documents. The map depicts 59 countries organised into seven distinct clusters, each represented by a unique colour. The size of each node correlates with the TLS metric, which measures the overall level of global collaboration between countries. A larger node size indicates a higher extent of international collaboration. The United States (TLS = 691), the United Kingdom (TLS = 462), Italy (TLS = 302), Belgium (TLS = 291), and Australia (TLS = 259) were the top five countries with the highest TLS (Figure 5). The thickness of the lines connecting the nodes represents the level of collaboration between countries. For example, the thickest connecting line indicates that the link strength between the United States and the United Kingdom is 88, the highest value of link strength between any two countries, signifying substantial collaboration between them. Conversely, the United States has the lowest link strength value of 1 with several countries, including Indonesia, Morocco, Romania, and Mexico, indicating minimal collaboration with these countries.

### Keyword Co-occurrence Analysis

**Figure 6:**
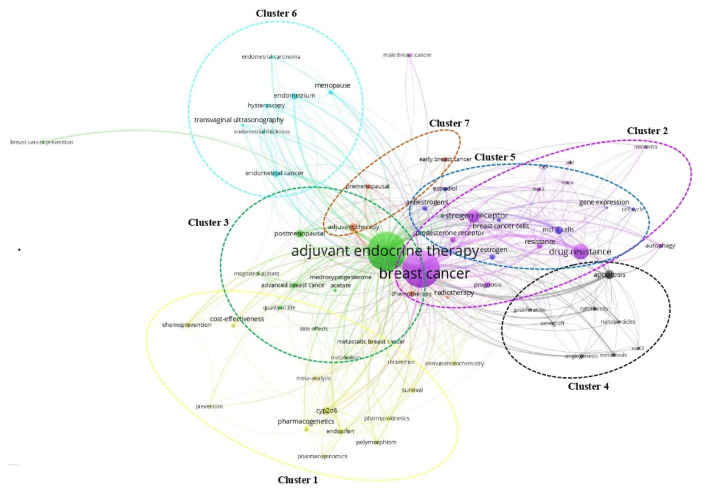
Co-occurrence network map of the 63 most used keywords related to research on tamoxifen application in treating breast cancer from 1973 to 2022

A keyword co-occurrence analysis was used to identify the hot-spot keywords involved in the research on tamoxifen application in treating breast cancer from 1973 to 2022. Out of the 3,660 author keywords, a mere 63 keywords exceeded the minimum threshold of 10 occurrences per keyword (Figure 6). From this analysis, seven different clusters were obtained. Every cluster represents a distinct sub-field of study about the research on tamoxifen application in treating breast cancer. Cluster 2, Cluster 3, and Cluster 1 are ranked as the top three clusters according to TLS metrics, occurrences, and average citations. Cluster 2, denoted by the purple circle, comprises protein kinases and receptors (human epidermal growth factor receptor 2 [HER2]), mTOR, epidermal growth factor receptor (EGFR), and Akt, all of which are proteins or kinases implicated in the pathogenesis of breast cancer via diverse cellular mechanisms. Subsequently, in Cluster 3, denoted by the green circle, terms including adjuvant endocrine therapy, medroxyprogesterone acetate, and megestrol acetate pertain to therapeutic modalities utilised in managing breast cancer patients. The keywords in Cluster 1 (shown by the yellow circle), such as pharmacogenetics, polymorphism, immunohistochemistry, pharmacokinetics, and pharmacogenomics, are the sub-research domains that have been actively explored in the field of breast cancer. Cluster 4 (shown by a black circle; keywords such as angiogenesis, apoptosis, metastasis, and proliferation), Cluster 5 (shown by a blue circle; keywords including cell cycle, oestradiol, and oestrogen), Cluster 6 (shown by turquoise circle; keywords such as endometrial cancer, hysteroscopy, and transvaginal ultrasonography), and Cluster 7 (shown by brown circle; keywords including adjuvant therapy, chemotherapy, and radiotherapy) are associated with cellular growth and regulation, hormonal regulation of cell cycle, diagnostic and management approaches for endometrial cancer, and systemic and local cancer treatments, respectively.

**Figure 7:**
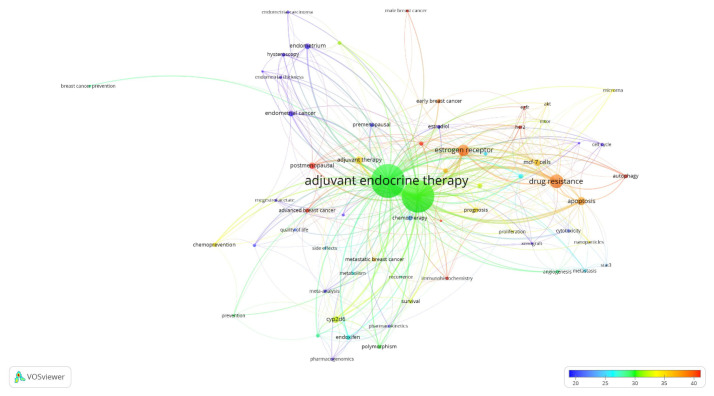
A network map overlay representation of keyword co-occurrences. The node size and colour scale represent the occurrences and the average number of citations

This study expands the visualisation presented in Figure 6 into an overlay visualisation to further analyse the research on the use of tamoxifen therapy in treating breast cancer (Figure 7). The top five keywords with over 80 occurrences reported from 1973 to 2022 are adjuvant endocrine therapy, breast cancer, drug resistance, oestrogen receptor, and apoptosis. For 50 years, the postmenopausal term had the highest average citation of 66.35, with EGFR, autophagy, and HER2 following closely after (Figure 7). The keyword “adjuvant endocrine therapy” had the highest TLS, link, and occurrences.

## Discussion

To the best of our knowledge, this is the first bibliometric study to focus specifically on research related to tamoxifen in breast cancer treatment. The study reveals a significant increase in research on the application of tamoxifen for treating breast cancer over the past 50 years. According to the inclusion criteria, 3,721 documents were analysed. Three thousand six hundred six authors affiliated with 11,915 organisations across 270 countries have contributed to this study. Over the past 50 years, the 3,721 documents on tamoxifen in breast cancer treatment have received 220,507 citations. The overall publishing trend exhibits a progressively polynomial pattern (R^2^ = 0.9099), indicating sustained global scholarly interest. Notably, the first paper on tamoxifen in breast cancer treatment, published in 1973, received the highest citations per cited paper. Older publications often receive more citations as foundational references for future research.

Researchers commonly employ *h*- and *g*-indices to measure their productivity and impact (18, 19). The *h*-index, however, lacks sensitivity to highly cited papers beyond the “*h*” value (20). The *g*-index was introduced in 2006, accounting for highly cited papers beyond the “*h*-value” or the “Hirsch core” (21). This makes the *g*-index more helpful in comparing researchers with similar *h*-index who may be competing for limited resources (22). We utilised both indices in this study to measure the research productivity and impact. For example, while the TP values in 2004 and 2005 were not the highest, these years had the most excellent *h*- and *g*-indices, indicating significant influence on subsequent studies. They either provided a foundation for future basic research or were used as references for more sophisticated and thorough studies.

Jordan, VC emerged as the leading researcher with the highest TP (*n* = 62) and *g*-index, reflecting his influential work. The *g*-index is a more precise measure as it considers the significance of the citations received by a scholar’s most influential publications and is not constrained by the total number of documents (23). Therefore, this indicator is more dependable and extensive in assessing a scholar’s efficiency and influence.

The United States led in TP output (*n* = 1,099), followed by the United Kingdom (*n* = 451). The United States and the United Kingdom were the most influential countries in tamoxifen and breast cancer research, likely due to substantial investments in research and development (R&D), advanced technologies, and supportive government policies. Morocco, a lower middle-income country, had the lowest values for TLS, links, and citations, likely due to economic constraints. Among organisations, those in Sweden dominated the top 10 rankings, suggesting high motivation and activity in tamoxifen and breast cancer research. This focus may have improved breast cancer survival rates and decreased mortality rates in Sweden (24). The parameters above are of paramount importance in ascertaining the prognosis of breast cancer, consequently impacting the approach to disease management that incorporates tried and true treatment modalities and interventions (25). The findings of the collaborative endeavour reveal that several prominent American organisations, including the Dana-Farber Cancer Institute, the Frontier Science and Technology Research Foundation, the National Cancer Institute, Fox Chase Cancer Centre, Memorial Sloan-Kettering Cancer Centre, Harvard Medical School, and the National Surgical Adjuvant Breast and Bowel Project (NSABP), also ranked highly, reflecting the substantial amount of human capital and technological advancement.

According to author keyword analysis co-occurrences, Clusters 1, 2, and 3 had the highest TLS values, specifically 2,819; 1,178; and 2,569, respectively. This suggests a significant level of interconnectedness among the items inside each cluster. As the result section emphasises, Cluster 1 embodies the actively explored sub-research domains within the breast cancer research landscape. This bibliometric analysis identified pharmacogenomics, pharmacogenetics, and polymorphism as active sub-research fields. According to this finding, these specific areas of research are essential for advancing precision medicine for breast cancer patients (26), enhancing the public health system by implementing preventive strategies (27) and uncovering genetic susceptibility (28). Cluster 2 is more pertinent to the pathogenesis of breast cancer. The development of breast cancer is influenced by a variety of pathways, such as the PI3K/Akt/mTOR, oestrogen, EGFR, mitogen-activated protein kinase (MAPK), p53, and JAK/STAT signalling pathways (29). EGFR, HER2, Akt, and mTOR are receptors/protein kinases with diverse roles in particular signalling pathways. For example, the activation of HER2 receptors triggers the signalling pathways of PI3K/Akt/mTOR and MAPK, which play a role in the development of breast cancer (30, 31). The primary focus of Cluster 3 is the application of hormone-based treatments, which are also known as endocrine therapy (ET), for the treatment of breast cancer. Tamoxifen, exemestane, anastrozole, letrozole, and fulvestrant are examples of ETs that have been utilised for the past three decades in the treatment of metastatic breast cancer that is positive for oestrogen receptors (ER-positive) (32). A plethora of clinical trials has been conducted to evaluate the efficacy and safety profile of ET, including ATLAS (Adjuvant Tamoxifen – Longer Against Shorter) (33), the NSABP B-42 trial (34), and the IDEAL trial (35). ET has been associated with several limitations, including the development of endometrial cancer, thromboembolic events, an increased risk of osteoporotic fractures, a decrease in bone mineral density, and myocardial infarction (36). Enhancing our knowledge of the underlying causes of breast cancer and utilising advanced technology in research could potentially overcome these constraints and provide a promising outlook for breast cancer patients in the future.

As stated in the results section, postmenopausal, EGFR, autophagy, and HER2 have garnered the highest average citations, suggesting that these keywords are focal points of research interest. Adjuvant ET was shown to be the most prominent and influential theme in this study, with the highest TLS, link, and occurrences. Postmenopausal women are defined as those aged 60 years or older, those who have undergone bilateral ovariectomy, or those younger than 60 years who have been amenorrhoeic for at least 12 months before the diagnosis of breast cancer and are not using hormone replacement therapy (HRT) or oral contraceptives while maintaining an intact uterus (37). Menopausal status is one of the critical factors in determining breast cancer treatment (37, 38). Guidelines from the National Comprehensive Cancer Network^®^ recommend tamoxifen or aromatase inhibitors as endocrine treatments for postmenopausal women diagnosed with early oestrogen receptor-positive (ER+) breast cancer (39). This explains the high citation rates for research on “postmenopausal” keywords.

Other keywords with high citations include EGFR and HER2. EGFR, part of the human epidermal receptor family, comprises four type I transmembrane tyrosine kinase receptors: EGFR (HER1, erbB-1), erbB-2 (HER2 or Neu), HER3, and HER4 (40). It plays significant roles in cell proliferation, survival (41), metastasis, angiogenesis (42), and treatment resistance (43). EGFR expression varies between breast cancer subtypes (44) and ethnic groups (45). Overexpression of EGFR is more common in triple-negative breast cancer (TNBC) compared to other subtypes (46, 47), and tamoxifen’s efficacy can depend on HER2 status (48).

Autophagy, another highly cited keyword, is a mechanism contributing to tamoxifen resistance (49, 50). Inhibiting autophagy can enhance the therapeutic efficacy of tamoxifen by overcoming endocrine resistance in ER+ breast cancers (51). “Adjuvant ET” also stands out due to its central role in tamoxifen-related breast cancer research (52). Adjuvant ET, including tamoxifen taken for five years, significantly reduces the long-term risk of recurrence and mortality in hormone receptor-positive, HER2-negative breast cancer subtypes (53). Apart from tamoxifen, other adjuvant endocrine therapies are summarised in Tables 6a–6c. Multiple studies have documented the use of tamoxifen and other endocrine therapies as switch therapy for the treatment of breast cancer patients (54–56).

## Limitation

To the best of our knowledge, this study is the first bibliometric analysis to highlight the application of tamoxifen in the treatment of breast cancer over the past 50 years. We have addressed all the research objectives, with a few exceptions. Nevertheless, this bibliometric analysis is subject to certain constraints. First, the Scopus database was the only source from which we retrieved articles for this investigation. We chose Scopus as our dataset due to its unparalleled comprehensiveness (81, 82). However, we may have overlooked some critical studies in other databases, including PubMed (83) and Web of Science and Dimensions (84). Furthermore, the study focuses exclusively on articles written in English, which may result in bias and the omission of critical studies on tamoxifen therapy in other languages. Finally, if the authors had not included our search query in their article titles, we may have missed out on specific articles relevant to tamoxifen therapy.

## Conclusions

This study is the first to use bibliometrics to analyse the application of tamoxifen in breast cancer treatment. It encompasses evaluations such as citation metrics, patterns of publication expansion, collaborations between countries and organisations on an international level, prolific authors, and research hot spots over time. The consistent annual increase in publication outputs indicates growing interest and clinical importance of this research field worldwide. The United States leads in publication output, followed closely by the United Kingdom. Over the past 50 years, key research foci have included adjuvant endocrine therapy, postmenopausal conditions, EGFR, autophagy, and HER2. These topics have attracted significant global attention due to their experimental and practical challenges.

## Figures and Tables

**Table 1:** The top 10 most productive authors contributed to papers on tamoxifen application for breast cancer

Author	TP	TC	NCP	*h*	*g*	Country
Jordan VC	62	6,498	60	34	62	United States
Dowsett M	50	10,650	50	37	50	United Kingdom
Stål O	43	2,055	43	25	43	Sweden
Foekens JA	41	2,755	41	30	41	Netherlands
Howell A	39	13,047	39	29	39	United Kingdom
Goldhirsch A	39	13,932	38	30	39	Switzerland/Norway
Nordenskjöld B	38	4,169	38	25	38	Sweden
Thürlimann B	38	14,151	38	29	38	Switzerland/United States
Wolmark N	37	27,619	37	30	37	United States
Cuzick J	37	15,794	36	29	37	United Kingdom

Notes: TP = total publications; TC = total citations; NCP = number of cited papers; *h* = h-index; *g* = g-index

**Table 2:** The top 10 authors ranked highest in TLS-based co-citation analysis

Rank	Author	Cluster	TLS	Citation	Country
1.	Jordan VC	3	38,818	2,254	United States
2.	Osborne CK	1	28,474	1,604	United States
3.	Dowsett M	1	25,684	1,252	United Kingdom
4.	Fisher B	3	20,613	1,450	United States
5.	Cuzick J	2	18,747	1,003	United Kingdom
6.	Howell A	2	17,736	808	United Kingdom
7.	Ingle JN	2	17,164	809	United States
8.	Desta Z	4	14,379	658	United States
9.	Rae JM	4	14,159	607	United States
10.	Nicholson RI	1	13,481	657	United Kingdom

**Table 3:** The top 10 organisations contributing to publications on the research of tamoxifen application in treating breast cancer

Organisation	Country	TP	*n* (%)	NCP	C/CP	TC	*h*
Erasmus MC	Netherlands	85	2.28	85	133.09	11,313	43
The University of Texas MD Anderson Cancer Center	United States	75	2.02	69	218.20	15,056	33
Karolinska Universitetssjukhuset	Sweden	69	1.85	68	132.01	8,977	36
National Cancer Institute	United States	68	1.83	66	349.35	23,057	39
Linköpings Universitet	Sweden	54	1.45	54	45.91	2,479	27
Dana-Farber Cancer Institute	United States	54	1.45	51	279.90	14,275	33
Cancer Research UK	United Kingdom	51	1.37	49	263.08	12,891	32
Karolinska Institutet	Sweden	50	1.34	49	43.76	2,144	26
Skånes Universitetssjukhus	Sweden	49	1.32	49	56.04	2,746	30
The Royal Marsden Hospital	United Kingdom	48	1.29	46	239.06	10,997	36

Notes: TP = total publications; *n* (%) = number in percentage; NCP = number of cited papers; C/CP = citations per cited paper; TC = total citations; *h* = h-index

**Table 4:** The most influential organisations in the research on tamoxifen application in treating breast cancer (ranking-based TLS)

Organisation	Country	TLS	Documents	Average citations
Swiss Groupfor Clinical Cancer Research (SAKK)	Switzerland	42	11	356.82
Dana-FarberCancerInstitute	United States	30	12	307.00
Frontier Scienceand Technology Research Foundation	United States	27	9	313.78
European Institute of Oncology	Italy	26	6	713.17
Oncology Institute of Southern Switzerland	Switzerland	26	7	323.86
University of Sydney	Australia	26	8	407.00
Breast Center, Kantonsspital St. Gallen	Switzerland	25	7	170.57
Department of Medicine, European Instituteof Oncology	Italy	18	5	125.80
International Breast Cancer Study Group	Switzerland	15	5	52.40
College of Medicine, China Medical University	Taiwan	12	7	13.86
Management Officef or Health Data, China Medical University Hospital	Taiwan	12	10	14.00
National Cancer Institute	United States	12	9	1,432.67
British Columbia Cancer Agency	Canada	11	8	864.63
College of Medicine, TzuChi University	Taiwan	10	5	15.60
Fox Chase Cancer Center,Philadelphia	United States	10	8	773.38
Memorial Sloan-Kettering Cancer Center	United States	10	5	823.60
Harvard Medical School	United States	9	6	43.17
Department of Oncology, Odense University Hospital	Denmark	7	12	44.58
National Surgical Adjuvant Breast and Bowel Project	United States	7	6	365.33
Department of Breast Oncology, International Medical Center, Saitama Medical University	Japan	5	5	42.00

**Table 5:** Top 10 of the most productive countries based on TP metric

Rank	Country	TP
1.	United States	1,099
2.	United Kingdom	451
3.	China	326
4.	Italy	263
5.	Japan	175
6.	Sweden	166
7.	Netherlands	165
8.	Canada	161
9.	Germany	155
10.	South Korea	137

**Table 6a:** An overview of the selective oestrogen receptor modulators (SERMs) offered to patients with hormone-dependent breast cancer

SERMs	Mechanism of action	Class	Regulatory status
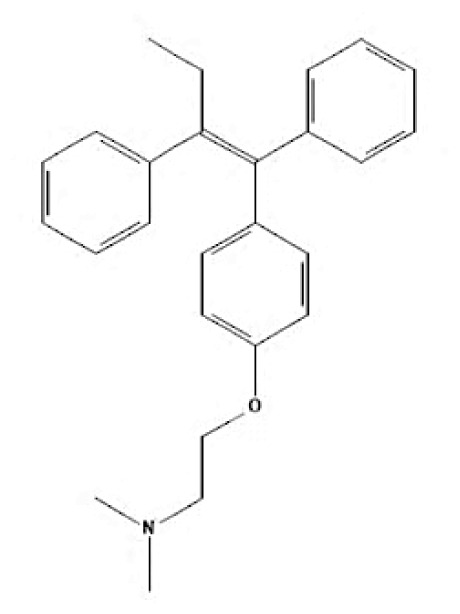 Tamoxifen	Due to its dual mechanism of action, it binds selectively to oestrogen receptors and generates both oestrogenic and anti-oestrogen effects (57).	First-generation non-steroidal SERMs	Approved by the FDA in 1977 for the treatment of breast cancer in both sexes (62).
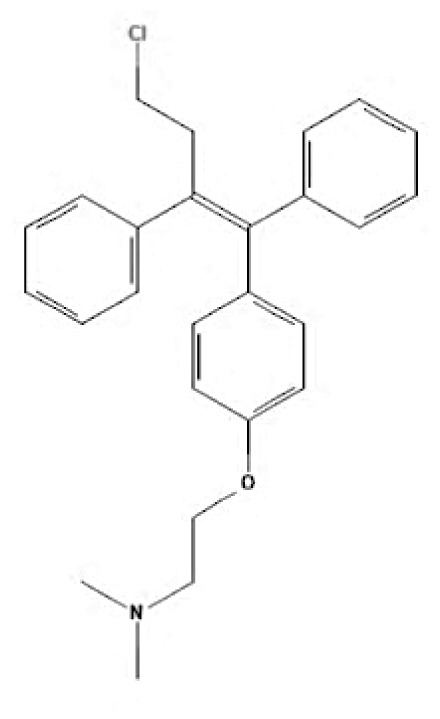 Toremifene	Has a similar mechanism to tamoxifen (58).	First-generation non-steroidal SERMs	FDA approved in 1995 for the treatment of advanced breast cancer (63). It was granted first-line treatment status by the FDA in 1997 for postmenopausal women diagnosed with oestrogen receptor (ER)-positive or unidentified metastatic breast cancer (64).
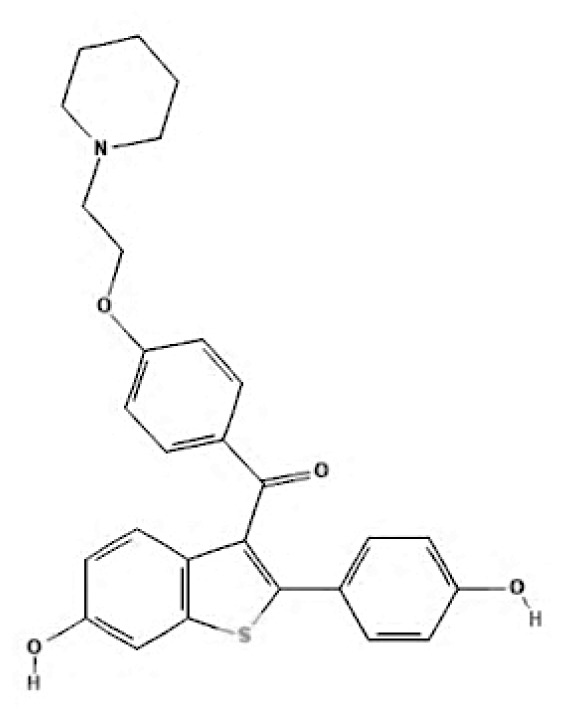 Raloxifene	Raloxifene exerts its mode of action by attaching to oestrogen receptors. Consequently, in tissues that express oestrogen receptors, the oestrogenic pathways (oestrogen agonistic effect) and blockade (oestrogen antagonistic effect) are brought into action (59).	Second-generation non-steroidal SERMs	Approved by the FDA in 2007 for the treatment and prevention of osteoporosis and cancer in postmenopausal individuals (65).
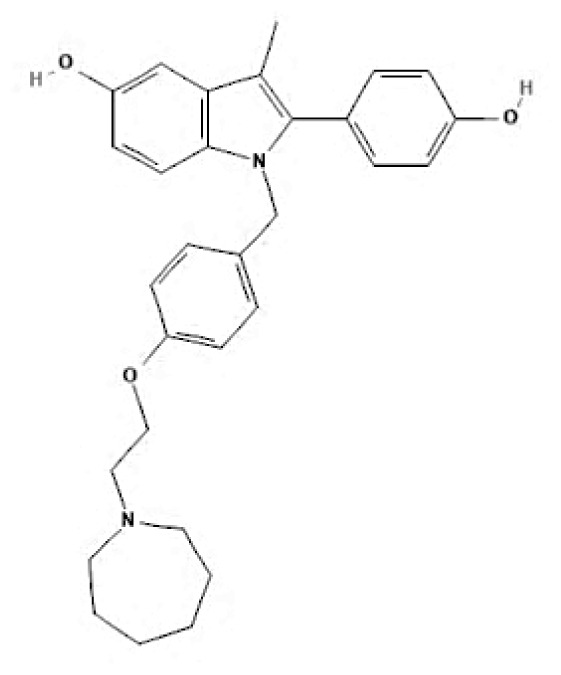 Bazedoxifene	Bazedoxifene exerts its mechanism of action via the oestrogen receptor or target tissue. It functions as a competitive antagonist, leading to antiproliferative actions in breast cancer. It also functions as an agonist in regulating bone turnover and stimulating lipid metabolism (60).	Third-generation non-steroidal SERMs	Bazedoxifene was approved by the FDA in 2013 as part of the combination drug Duavee, which is used to prevent postmenopausal osteoporosis and manage vasomotor symptoms associated with menopause (66).
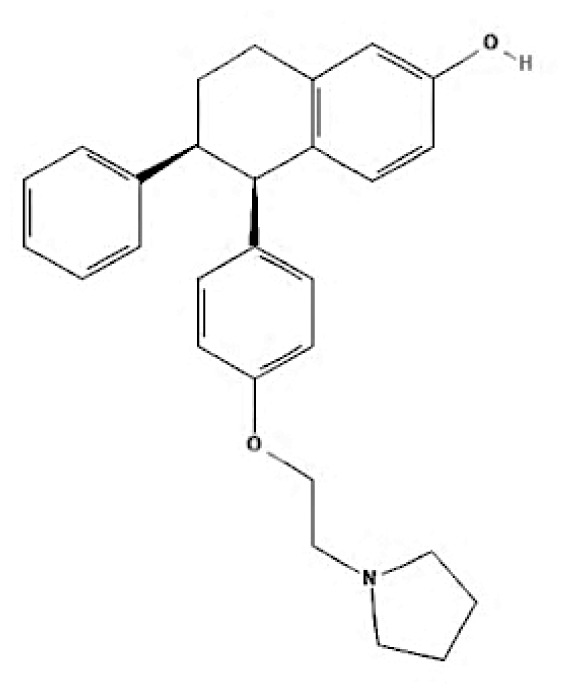 Lasofoxifene	It binds selectively and with an affinity comparable to oestradiol to human ERα and Erβ (61).	Third-generation non-steroidal SERMs	In 2009, the European Union approved the use of lasofoxifene specifically for postmenopausal women with osteoporosis who have a higher risk of experiencing fractures (67).

**Table 6b:** A summary of selective oestrogen receptor downregulators or degraders (SERDs) available for patients with hormone-dependent breast cancer

SERDs	Mechanism of action	Class	FDA approval
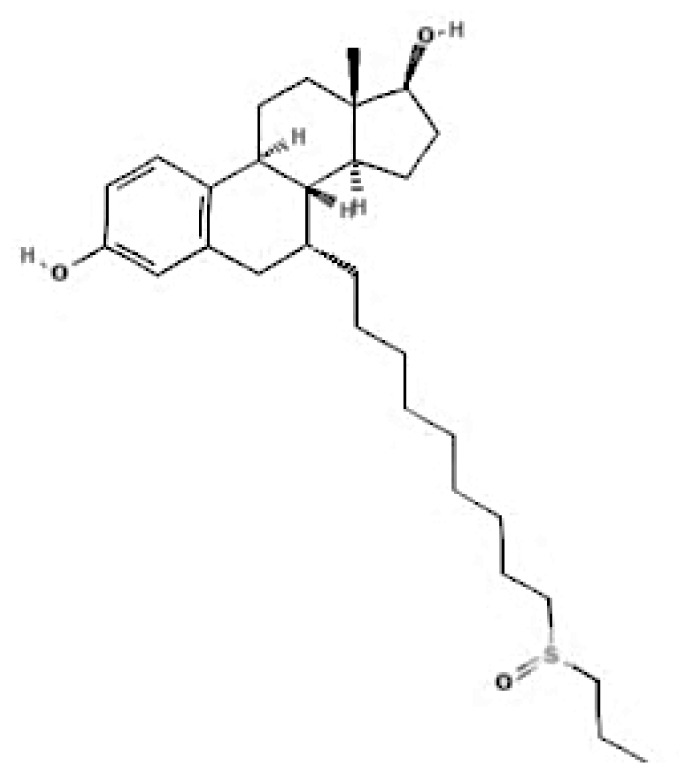 Fulvestrant	It exerts its antagonistic effects through direct binding to the oestrogen receptor. When fulvestrant induces a conformational change in the ER, AF2—and AF1-related transcriptional activities are disrupted (68). The process by which fulvestrant binds to the oestrogen receptor induces the creation of an unstable complex, subsequently leading to an expedited degradation of the oestrogen receptor protein (69). Hence, by this binding, oestrogen signalling via the oestrogen receptor can be entirely inhibited (70).	Steroidal antioestrogen	In 2002, it received FDA approval for monthly intramuscular injection as a treatment for hormone receptor-positive metastatic breast cancer in postmenopausal women who have experienced disease progression after antioestrogen therapy (71). In 2007, the FDA approved the use of this medication to treat metastatic luminal breast cancer in postmenopausal patients who had progressed on aromatase inhibitors or tamoxifen-based ET (72).

**Table 6c:** A comprehensive overview of several generations of aromatase inhibitors (AIs) available for patients with hormone-dependent breast cancer

Aromatase inhibitors (AIs) The first-generation of AIs	Mechanism of action	Class	Regulatory status
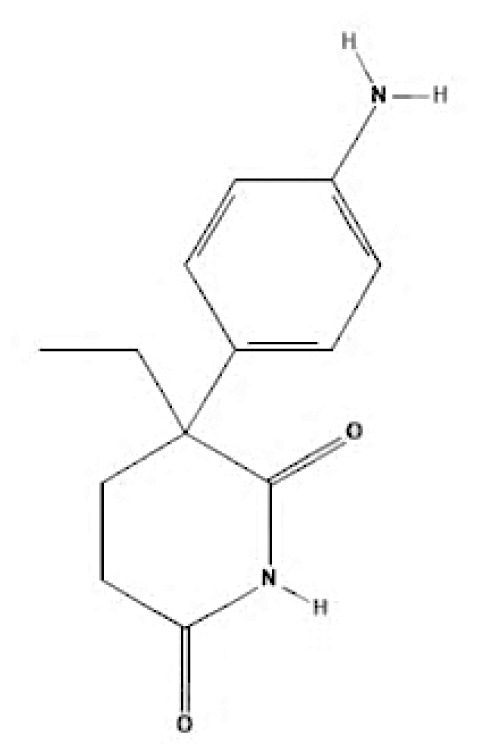 Aminoglutethimide	Aminoglutethimide has been proposed to have several potential mechanisms of action, including aromatase inhibition (73), medical adrenalectomy (74), and increased oestrogen metabolism (75).	Non-steroidal	In 1960, it received FDA approval for the treatment of breast cancer. However, it was later pulled from the market due to adrenal insufficiency (76).
**Second-generation of AIs**	**Mechanism of action**	**Class**	**Regulatory status**
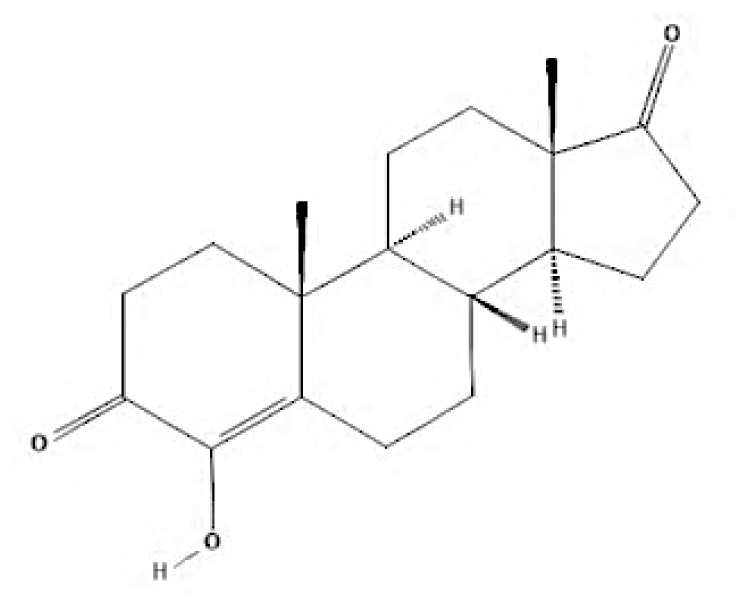 Formestane	It forms a covalent bond with the substrate-binding site of aromatase, permanently inactivating it (77).	Type I (steroidal) aromatase inhibitor	-
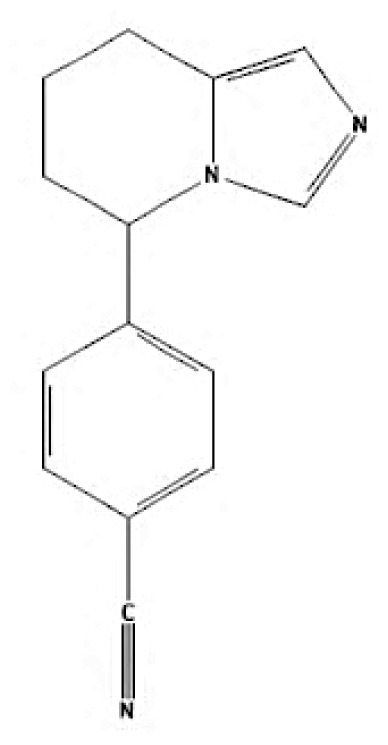 Fadrozole	NSAIDs form a non-covalent bond with the haem of aromatase, effectively blocking the androgen from attaching to the catalytic site. This binding exerts a reversible inhibitory effect on aromatase (77).	Type II (non-steroidal) aromatase inhibitor	-
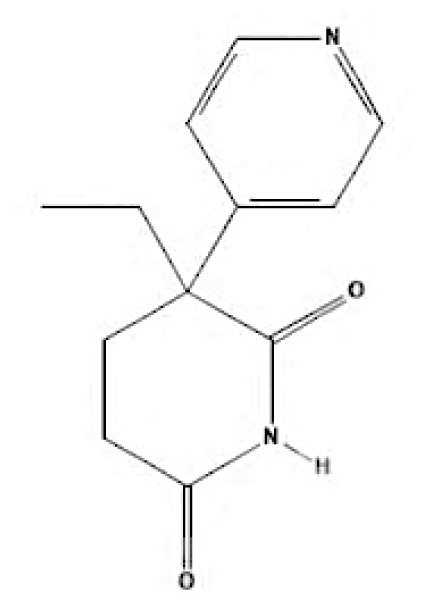 Rogletimide	NSAIDs form a non-covalent bond with the haem of aromatase, effectively blocking the androgen from attaching to the catalytic site. This binding exerts a reversible inhibitory effect on aromatase (77).	Type II (non-steroidal) aromatase inhibitor	-
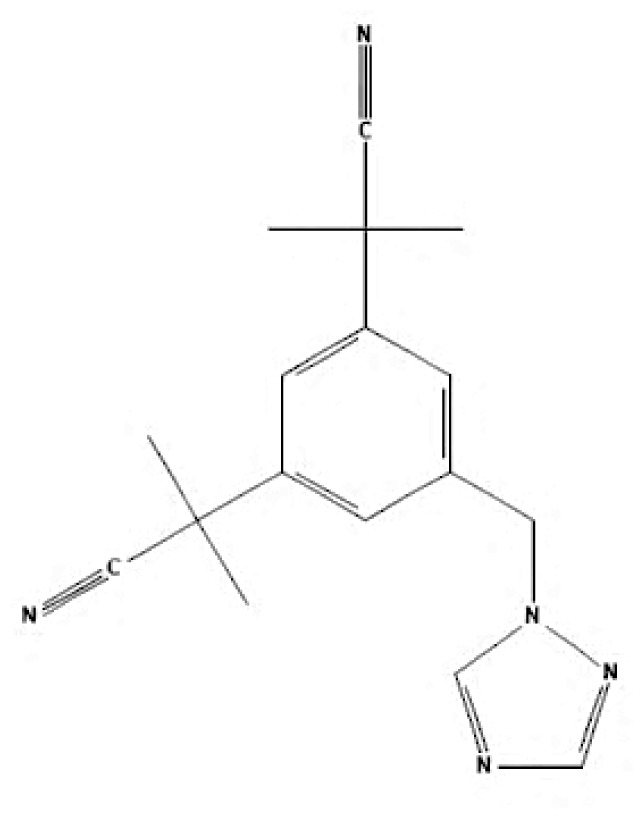 Anastrozole	NSAIDs form a non-covalent bond with the haem of aromatase, effectively blocking the androgen from attaching to the catalytic site. This binding exerts a reversible inhibitory effect on aromatase (77).	Type II (non-steroidal) aromatase inhibitor	Received FDA approval in 1996 for treating advanced breast cancer in postmenopausal women who had experienced disease progression after tamoxifen therapy (78).
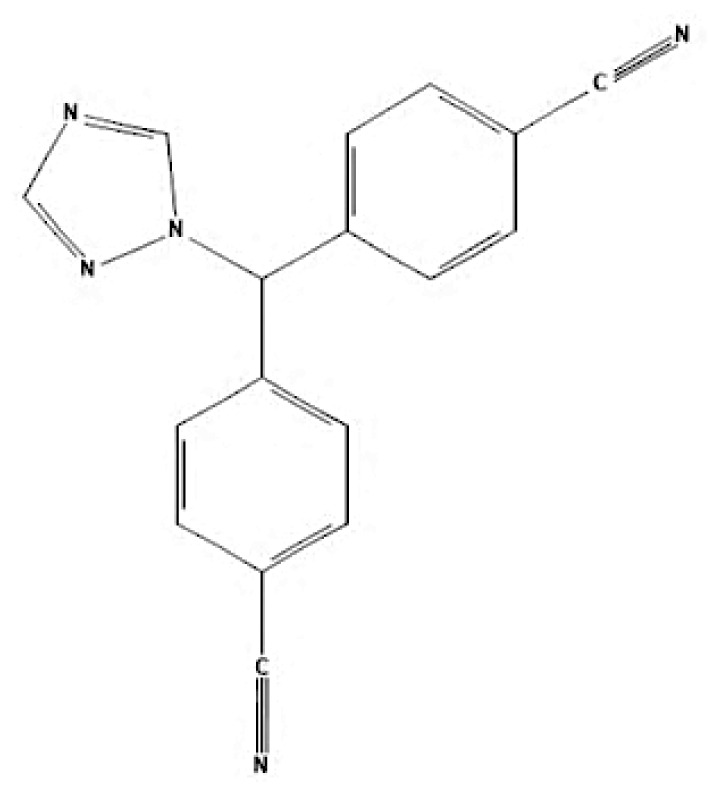 Letrozole	NSAIDs form a non-covalent bond with the haem of aromatase, effectively blocking the androgen from attaching to the catalytic site. This binding exerts a reversible inhibitory effect on aromatase (77).	Type II (non-steroidal) aromatase inhibitor	Acquired approval from the FDA in 1998 for the treatment of advanced breast cancer in postmenopausal women who had previously failed one antioestrogen treatment and had hormone receptor-positive or unknown breast cancer (79).
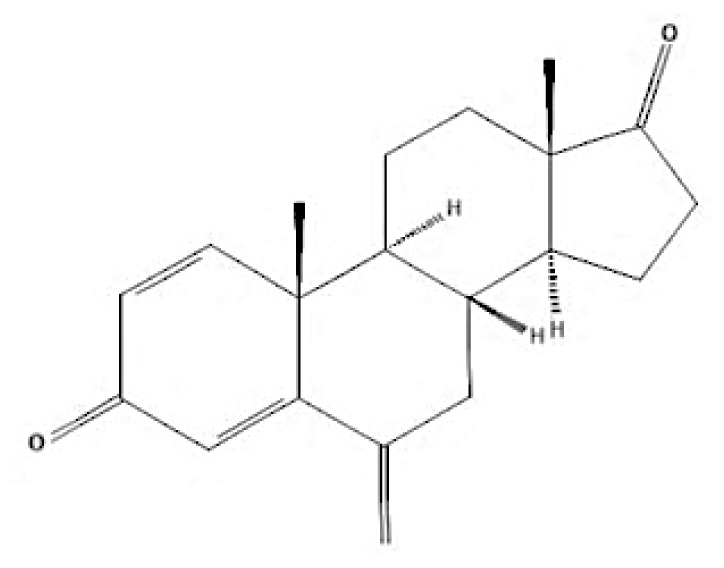 Exemestane	It forms a covalent bond with the substrate-binding site of aromatase, permanently inactivating it (77).	Type I (steroidal aromatase) inhibitor	Received FDA approval in 1999 for use as an adjuvant treatment in postmenopausal women with early breast cancer who have oestrogen receptor-positive tumours. This treatment is intended for those who have already taken tamoxifen for 2–3 years and need to complete a total of five consecutive years of hormonal therapy (80).

## References

[1] Ferlay J, Ervik M, Lam F, Laversanne M, Colombet M, Mery L (2024). Global cancer observatory: cancer today [Internet].

[2] Lopes CCC (2022). Correlation between the incidence of breast cancer and the human development index. Ame J Surg Clin Case Rep.

[3] Ghoncheh M, Mohammadian-Hafshejani A, Salehiniya H (2015). Incidence and mortality of breast cancer and their relationship to development in Asia. Asian Pac J Cancer Prev.

[4] Cantini L, Bertoli G, Cava C, Dubois T, Zinovyev A, Caselle M (2019). Identification of microRNA clusters cooperatively acting on epithelial to mesenchymal transition in triple negative breast cancer. Nucleic Acids Res.

[5] Dai X, Li T, Bai Z, Yang Y, Liu X, Zhan J (2015). Breast cancer intrinsic subtype classification, clinical use and future trends. Am J Cancer Res.

[6] Yersal O, Barutca S (2014). Biological subtypes of breast cancer: Prognostic and therapeutic implications. World J Clin Oncol.

[7] WHO Classification of Tumours Editorial Board (2019). Breast tumours.

[8] Saraiva D, Cabral MG, Jacinto A, Braga S (2017). How many diseases is triple negative breast cancer: the protagonism of the immune microenvironment. Esmo Open.

[9] Puppe J, Seifert T, Eichler C, Pilch H, Mallmann P, Malter W (2020). Genomic signatures in luminal breast cancer. Breast Care (Basel).

[10] Baumann CK, Castiglione-Gertsch M (2009). Clinical use of selective estrogen receptor modulators and down regulators with the main focus on breast cancer. Minerva Ginecol.

[11] Howell A, Howell SJ (2023). Tamoxifen evolution. Br J Cancer.

[12] Matariek G, Teibo JO, Elsamman K, Teibo TK, Olatunji DI, Matareek A (2022). Tamoxifen: the past, present, and future of a previous orphan drug. European Journal of Medical and Health Sciences.

[13] Page MJ, McKenzie JE, Bossuyt PM, Boutron I, Hoffmann TC, Mulrow CD (2021). The PRISMA 2020 statement: an updated guideline for reporting systematic reviews. BMJ.

[14] Arruda H, Silva ER, Lessa M, Proença D, Bartholo R (2022). VOSviewer and bibliometrix. J Med Libr Assoc.

[15] Martín-Martín A, Thelwall M, Orduna-Malea E, Delgado López-Cózar E (2021). Google Scholar, Microsoft Academic, Scopus, Dimensions, Web of Science, and OpenCitations’ COCI: a multidisciplinary comparison of coverage via citations. Scientometrics.

[16] Ward HW (1973). Anti-oestrogen therapy for breast cancer: a trial of tamoxifen at two dose levels. Br Med J.

[17] (2024). UNDP, Human Development Report (2024) – with minor processing by Our World in Data. “Human Development Index groups” [dataset]. UNDP, Human Development Report, “Human Development Report 2023–2024 ” [original data] [Internet].

[18] Mondal H, Deepak KK, Gupta M, Kumar R (2023). The *h*-Index: Understanding its predictors, significance, and criticism. J Family Med Prim Care.

[19] Ali MJ (2021). Understanding the “*g*-index”and the “*e*-index”. Semin Ophthalmol.

[20] Ali MJ (2021). Forewarned is forearmed: the *h*-index as a scientometric. Semin Ophthalmol.

[21] Roldan-Valadez E, Salazar-Ruiz SY, Ibarra-Contreras R, Rios C (2019). Current concepts on bibliometrics: a brief review about impact factor, Eigenfactor score, CiteScore, SCImago Journal Rank, Source-Normalised Impact per Paper, *H*-index, and alternative metrics. Ir J Med Sci.

[22] Ahangar HG, Siamian H, Yaminfirooz M (2014). Evaluation of the scientific outputs of researchers with similar h index: a critical approach. Acta Inform Med.

[23] Costas R, Bordons M (2008). Is *g*-index better than *h*-index? An exploratory study at the individual level. Scientometrics.

[24] Roginski M, Sifaki-Pistolla D, Stomby A, Velivasaki G, Faresjö T, Lionis C (2022). Paradoxes of breast cancer incidence and mortality in two corners of Europe. BMC Cancer.

[25] WHO Essential Medicines List eEML - Electronic Essential Medicines List. [Internet].

[26] Sarhangi N, Hajjari S, Heydari SF, Ganjizadeh M, Rouhollah F, Hasanzad M (2022). Breast cancer in the era of precision medicine. Molecular Biology Reports.

[27] Karbakhsh M (2021). Global Breast Cancer Initiative: an integrative approach to thinking globally, acting locally. Archives of Breast Cancer.

[28] Valentini V, Bucalo A, Conti G, Celli L, Porzio V, Capalbo C (2024). Gender-specific genetic predisposition to breast cancer: BRCA genes and beyond. Cancers.

[29] Toss A, Venturelli M, Peterle C, Piacentini F, Cascinu S, Cortesi L (2017). Molecular biomarkers for prediction of targeted therapy response in metastatic breast cancer: trick or treat?. International Journal of Molecular Sciences.

[30] Wee P, Wang Z (2017). Epidermal growth factor receptor cell proliferation signaling pathways. Cancers.

[31] Elizalde PV, Russo RIC, Chervo MF, Schillaci R (2016). ErbB-2 nuclear function in breast cancer growth, metastasis and resistance to therapy. Endocrine-Related Cancer.

[32] Patel R, Klein P, Tiersten A, Sparano JA (2023). An emerging generation of endocrine therapies in breast cancer: a clinical perspective. NPJ Breast Cancer.

[33] Davies C, Pan H, Godwin J, Gray R, Arriagada R, Raina V (2013). Long-term effects of continuing adjuvant tamoxifen to 10 years versus stopping at 5 years after diagnosis of oestrogen receptor-positive breast cancer: ATLAS, a randomised trial. The Lancet.

[34] Mamounas EP, Bandos H, Lembersky BC, Jeong JH, Geyer CE, Rastogi P (2019). Use of letrozole after aromatase inhibitor-based therapy in postmenopausal breast cancer (NRG Oncology/NSABP B-42): a randomised, double-blind, placebo-controlled, phase 3 trial. The Lancet Oncology.

[35] Blok EJ, Kroep JR, Meershoek-Klein Kranenbarg E, Duijm-de Carpentier M, Putter H, van den Bosch J (2018). Optimal duration of extended adjuvant endocrine therapy for early breast cancer; results of the IDEAL trial (BOOG 2006–05). Journal of the National Cancer Institute.

[36] Matthews A, Stanway S, Farmer RE, Strongman H, Thomas S, Lyon AR (2018). Long term adjuvant endocrine therapy and risk of cardiovascular disease in female breast cancer survivors: systematic review. BMJ.

[37] De Vos FY, van Laarhoven HW, Laven JS, Themmen AP, Beex LV, Sweep CG (2012). Menopausal status and adjuvant hormonal therapy for breast cancer patients: a practical guideline. Critical Reviews in Oncology/Hematology.

[38] Bernard-Marty C, Cardoso F, Piccart MJ (2004). Facts and controversies in systemic treatment of metastatic breast cancer. Oncologist.

[39] National Comprehensive Cancer Network (2019). NCCN Clinical Practice Guidelines in Oncology (NCCN Guidelines^®^) Breast Cancer Version 1.

[40] Zık B, Asmaz ED (2018). Effect of tamoxifen treatment on the epidermal growth factor receptor expression in the mouse ovarian tissue. Uludağ Üniversitesi Veteriner Fakültesi Dergisi.

[41] Song X, Liu Z, Yu Z (2020). EGFR promotes the development of triple negative breast cancer through JAK/STAT3 signaling. Cancer Manag Res.

[42] Citri A, Yarden Y (2006). EGF–ERBB signalling: towards the systems level. Nat Rev Mol Cell Biol.

[43] Byeon HK, Ku M, Yang J (2019). Beyond EGFR inhibition: multilateral combat strategies to stop the progression of head and neck cancer. Exp Mol Med.

[44] Opstal-van Winden AW, Krop EJ, Kåredal MH, Gast MC, Lindh CH, Jeppsson MC (2011). Searching for early breast cancer biomarkers by serum protein profiling of pre-diagnostic serum; a nested case-control study. BMC Cancer.

[45] Zhang L, Fang C, Xu X, Li A, Cai Q, Long X (2015). Androgen receptor, EGFR, and BRCA1 as biomarkers in triple-negative breast cancer: a meta-analysis. Biomed Res Int.

[46] Masuda H, Zhang D, Bartholomeusz C, Doihara H, Hortobagyi GN, Ueno NT (2012). Role of epidermal growth factor receptor in breast cancer. Breast Cancer Res Treat.

[47] Lehmann BD, Bauer JA, Chen X, Sanders ME, Chakravarthy AB, Shyr Y (2011). Identification of human triple-negative breast cancer subtypes and preclinical models for selection of targeted therapies. J Clin Invest.

[48] Mansouri S, Mokhtari-Hesari P, Naghavi-Al-Hosseini F, Seyednejad SA, Majidzadeh-A K, Moradi-Kalbolandi S (2021). Evaluating human epidermal growth factor receptor 2 roles in the efficacy of Tamoxifen treatment in breast cancer, a systematic review. Pharmacol Rep.

[49] Samaddar JS, Gaddy VT, Duplantier J, Thandavan SP, Shah M, Smith MJ (2008). A role for macroautophagy in protection against 4-hydroxytamoxifen–induced cell death and the development of antiestrogen resistance. Mol Cancer Ther.

[50] Qadir MA, Kwok B, Dragowska WH, To KH, Le D, Bally MB (2008). Macroautophagy inhibition sensitizes tamoxifen-resistant breast cancer cells and enhances mitochondrial depolarization. Breast Cancer Res Treat.

[51] Liu J, Yue W, Chen H (2019). The correlation between autophagy and tamoxifen resistance in breast cancer. Int J Clin Exp Pathol.

[52] Waks AG, Winer EP (2019). Breast cancer treatment: a review. JAMA.

[53] Early Breast Cancer Trialists’ Collaborative Group (2011). Relevance of breast cancer hormone receptors and other factors to the efficacy of adjuvant tamoxifen: patient-level meta-analysis of randomised trials. Lancet.

[54] Butani D, Gupta N, Jyani G, Bahuguna P, Kapoor R, Prinja S (2021). Cost-effectiveness of tamoxifen, aromatase inhibitor, and switch therapy (adjuvant endocrine therapy) for breast cancer in hormone receptor positive postmenopausal women in India. Breast Cancer: Targets and Therapy.

[55] Gierach GL, Curtis RE, Pfeiffer RM, Mullooly M, Ntowe EA, Hoover RN (2017). Adjuvant Endocrine Therapy and Risk of Contralateral Breast Cancer among US Women with Breast Cancer. JAMA Oncology.

[56] Morden JP, Alvarez I, Bertelli G, Coates AS, Coleman R, Fallowfield L (2017). Long-term follow-up of the intergroup exemestane study. Journal of Clinical Oncology.

[57] Farrar MC, Jacobs TF (2023). Tamoxifen. StatPearls [Internet].

[58] Mao C, Yang ZY, He BF, Liu S, Zhou JH, Luo RC (2012). Toremifene versus tamoxifen for advanced breast cancer. Cochrane Database Syst Rev.

[59] Quintanilla Rodriguez BS, Correa R (2023). Raloxifene. StatPearls [Internet].

[60] Gennari L, Merlotti D, De Paola V, Martini G, Nuti R (2008). Bazedoxifene for the prevention of postmenopausal osteoporosis. Ther Clin Risk Manag.

[61] Ke HZ, Qi H, Crawford DT, Chidsey-Frink KL, Simmons HA, Thompson DD (2000). Lasofoxifene (CP-336,156), a selective estrogen receptor modulator, prevents bone loss induced by aging and orchidectomy in the adult rat. Endocrinology.

[62] Eggemann H, Altmann U, Costa SD, Ignatov A (2018). Survival benefit of tamoxifen and aromatase inhibitor in male and female breast cancer. J Cancer Res Clin Oncol.

[63] Arora A, Potter JF (2004). Aromatase inhibitors: current indications and future prospects for treatment of postmenopausal breast cancer. J Am Geriatr Soc.

[64] Vogel CL (1998). Phase II and III clinical trials of toremifene for metastatic breast cancer. Oncology (Williston Park).

[65] Hong S, Chang J, Jeong K, Lee W (2021). Raloxifene as a treatment option for viral infections. J Microbiol.

[66] Raina PM, Parmar M (2023). Bazedoxifene. StatPearls [Internet].

[67] Grogan K (2009). Pfizer and Ligand celebrate European approval of Fablyn [Internet].

[68] Movérare-Skrtic S, Börjesson AE, Farman HH, Sjögren K, Windahl SH, Lagerquist MK (2014). The estrogen receptor antagonist ICI 182,780 can act both as an agonist and an inverse agonist when estrogen receptor α AF-2 is modified. Proc Natl Acad Sci U S A.

[69] Nicholson RI, Gee JM, Manning DL, Wakeling AE, Montano MM, Katzenellenbogen BS (1995). Responses to Pure Antiestrogens (ICI 164384, ICI182780) in Estrogen-Sensitive and-Resistant Experimental and Clinical Breast Cancer. Ann N Y Acad Sci.

[70] Osborne CK, Wakeling A, Nicholson RI (2004). Fulvestrant: an oestrogen receptor antagonist with a novel mechanism of action. Br J Cancer.

[71] Bross PF, Cohen MH, Williams GA, Pazdur R (2002). FDA drug approval summaries: fulvestrant. Oncologist.

[72] Robertson JF, Lindemann J, Garnett S, Anderson E, Nicholson RI, Kuter I (2014). A good drug made better: the fulvestrant dose-response story. Clin Breast Cancer.

[73] Santen RJ, Santner S, Davis B, Veldhuis J, Samojlik E, Ruby E (1978). Aminoglutethimide inhibits extraglandular estrogen production in postmenopausal women with breast carcinoma. J Clin Endocrinol Metab.

[74] Griffiths CT, Hall TC, Saba Z, Barlow JJ, Nevinny HB (1973). Preliminary trial of aminoglutethimide in breast cancer. Cancer.

[75] Lønning PE, Kvinnsland S, Thorsen T, Ueland PM (1987). Alterations in the metabolism of oestrogens during treatment with aminoglutethimide in breast cancer patients: preliminary findings. Clin Pharmacokinet.

[76] Avendaño C, Menéndez JC (2015). Anticancer drugs that modulate hormone action: anticancer drugs that modulate hormone action. Medicinal chemistry of anticancer drugs.

[77] Chumsri S, Howes T, Bao T, Sabnis G, Brodie A (2011). Aromatase, aromatase inhibitors, and breast cancer. J Steroid Biochem Mol Biol.

[78] Knoche AJ, Michaud LB, Buzdar AU (1999). Efficacy of anastrozole in a consecutive series of advanced breast cancer patients treated with multiple prior chemotherapies and endocrine agents: MD Anderson Cancer Center experience. Breast J.

[79] Cohen MH, Johnson JR, Li N, Chen G, Pazdur R (2002). Approval summary: letrozole in the treatment of postmenopausal women with advanced breast cancer. Clin Cancer Res.

[80] Hashemi-Meshkini A, Keshavarz K, Gharibnaseri Z, Kheirandish M, Kebriaeezadeh A, Nikfar S (2013). Cost-effectiveness analysis review of exemestane in the treatment of primary and advanced breast cancer. Arch Med Sci.

[81] Pranckutė R (2021). Web of Science (WoS) and Scopus: The titans of bibliographic information in today’s academic world. Publications.

[82] Zhu J, Liu W (2020). A tale of two databases: The use of Web of Science and Scopus in academic papers. Scientometrics.

[83] Sayers EW, Bolton EE, Brister JR, Canese K, Chan J, Comeau DC, Connor R, Funk K, Kelly C, Kim S, Madej T (2022). Database resources of the national center for biotechnology information. Nucleic Acids Res.

[84] Singh VK, Singh P, Karmakar M, Leta J, Mayr P (2021). The journal coverage of Web of Science, Scopus and Dimensions: A comparative analysis. Scientometrics.

